# Seroepidemiological study of the exposure to *Toxoplasma gondii* among horses in Algeria and analysis of risk factors

**DOI:** 10.14202/vetworld.2019.2007-2016

**Published:** 2019-12-20

**Authors:** Sabrine Fazia Ouslimani, Safia Tennah, Naouelle Azzag, Salima Yamina Derdour, Bernard China, Farida Ghalmi

**Affiliations:** 1 Research Laboratory Management of Local Animal Resources, Higher National Veterinary School of Algiers, Oued Smar, Algiers 16000, Algeria; 2 Sciensano (Research Institute and The National Public Health Institute of Belgium), Juliette Wytsman street 14, 1050 Brussels, Belgium

**Keywords:** Algeria, horse, seroprevalence, *Toxoplasma gondii*

## Abstract

**Aim:**

The aim of this study was to assess the seroprevalence of the *Toxoplasma gondii* in horses in different parts of Algeria and to determine risk factors for the infection.

**Materials and Methods:**

A total of 736 blood samples were collected from horses of various breeds, gender, coat colors, and ages. All horses came from various farms, racecourses, and equestrian centers. The seroprevalence was investigated by three different methods: Indirect fluorescent antibody test (IFAT) as reference method, enzyme-linked immunosorbent assay (ELISA), and latex agglutination test (LAT).

**Results:**

Out of the 736 sera, 178 (24.18%) were positive for IFAT, 133 (18.07%) for LAT, and 317 (43.07%) for ELISA. It was found that IFAT and LAT were in high agreement (Kappa 0.79), indicating that LAT and IFAT had similar capabilities in the detection of anti-*T. gondii* antibodies from horse sera. Risk factors analysis based on IFAT results indicated that the habit of the animals was significant risk factors (p≤0.05) for *Toxoplasma* infection. The seroprevalence was significantly higher in horses living on farms. Moreover, a higher seroprevalence was found in older animals compared to younger ones. Furthermore, the seroprevalence in females was significantly higher than that in males and gelding. Breed, coat color, and water sources are also important factors to influence the seroprevalence of *T. gondii*.

**Conclusion:**

The results indicated that *T. gondii* is present in horses throughout Algeria and thus represents a risk for both human and animal health. These results underline the need to increase the vigilance and the preventive measures against this disease not only to protect the horses but also to limit the spread of the parasite.

## Introduction

Toxoplasmosis is a worldwide reported zoonotic infection caused by the protozoon *Toxoplasma gondii*. The obligate intracellular parasite infects a broad range of mammalian and avian hosts including approximately one-third of the human population [1]. In this zoonosis, the definitive host of *T. gondii* is the cat and related wild felids; these are the only hosts shedding oocysts into the environment to contaminate pastures, food items, and water [2]. A single cat can pass more than 100 million non-sporulated oocysts, which become infective within 1-5 days after [3]. *T. gondii* forms cysts in host tissues, and infection is considered to be lifelong [4]. Oocysts are central in the life cycle of *T. gondii*. After one up to a few days of maturation (sporulation) in the environment, they become infective to a large variety of warm-blooded intermediate hosts if ingested. In addition to oocysts, there are two further stages of *T. gondii*, which are infective, i.e., tachyzoites and bradyzoites, the latter being present in tissue cysts. After infection, tachyzoites invade host cells, in which they multiply. This replication is strictly intracytoplasmatic in parasitophorous vacuoles formed by the parasite. In parallel, after several rounds of multiplication; the parasite establishes intracellular tissue cysts, which contain slowly or no longer replicating bradyzoites. Tissue cyst formation preferentially occurs in brain tissue, the skeletal and heart muscle or also in the retina of infected intermediate hosts [5]. The parasite has a complex lifecycle, and multiple routes of infection are possible [2]. Humans get infected after ingesting undercooked or raw meat, by ingesting cat-shed oocysts through contaminated soil, food, water, or congenitally by transplacental transmission of tachyzoites [6]. Herbivores acquire infection generally by the ingestion of oocysts, shed by infected cats, in water, or contaminated food [7]. Infection with *T. gondii* in domestic equids has been reported in many countries [8]. Horses are most commonly infected by ingestion of sporulated oocysts found in feces of infected cats [9]. The infection is subclinical, however, atypical clinical signs of toxoplasmosis such as fever, ataxia, retinal degeneration, and encephalomyelitis may develop in horses [10].

Conventionally, in some areas of Algeria, uncooked horse meat is recommended for convalescence and anemia, which increases the risk of *T. gondii* infection. It is, therefore, important to know the occurrence of *T. gondii* in horses to prevent zoonotic infections by infected meat. In Algeria, there is a lack of information on infection of *T. gondii* in horses. In the present study, to elucidate the seroprevalence of *T. gondii* in horses in Algeria, we evaluated the sensitivity of commercial tests latex agglutination test (LAT) and enzyme-linked immunosorbent assay (ELISA), in comparison to in-house serologic reference test (indirect fluorescent antibody test [IFAT]) in the detection of specific antibodies against *T. gondii* in horses.

The aim of this study was to assess the seroprevalence of the *Toxoplasma gondii* in horses in different parts of Algeria and to determine risk factors for the infection.

## Materials and Methods

### Ethical approval and informed consent

This study was approved by the scientific council of High National Veterinary School, Algiers, Algeria. Informed consent was obtained from all the participants.

### Sampling plan and area study

**Figure-1:**
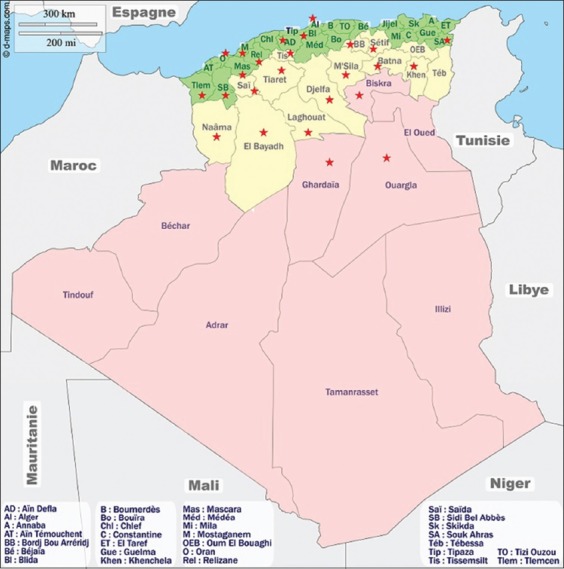
Map of Algeria on which are visible the three main regions according to our study. The stars indicate the regions in which the horses were taken [Source: d-maps.com (https://d-maps.com/carte.php?num_car=4428&lang=fr)].

Algeria is a large country (2,000,000 km^2^) consisting of three main regions and 48 departments (governorates) (Figure-1).

- Northern Algeria (tell) which has 25 governorates, 4% of the territory and 63% of the population;
- The highlands which have 14 governorates, 9% of the territory and 28% of the population; our study;
- The South or Sahara: This has nine governorates, 87% of the territory and 9% of the population [11].

To sample such a large area, the sample size was calculated according to:

N=1.96^2^ P (1−P)/D^2^

Where: N=Size of the sample; P: Expected prevalence; D: Required precision.

Following the data from Mohamed-Cherif *et al*. [12] on horses in Tiaret (Algeria), the expected prevalence of toxoplasmosis in Algeria is 26% and the desired absolute precision is 5%.

Therefore, the following Thrusfield [12], N=292.

To increase the absolute precision (D=3%), we randomly selected a sample of 736 individuals.

**Figure-2:**
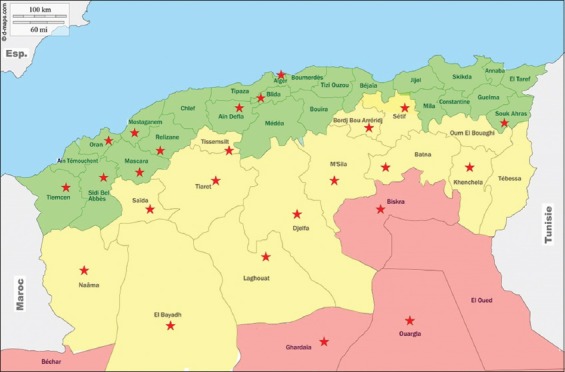
Map of Algeria from where samples collected. Stars indicates the location sampled for horses [Source: d-maps.com (https://d-maps.com/carte.php?num_car=798&lang=fr)].

The areas we have targeted correspond to regions/governorates with a high horse population. We have selected ten governorates in the region north, 12 in the highlands, and three in the region south. They are mentioned by stars in Figures-1 and 2.

### Blood sampling

Blood samples were obtained from each of the *Equidae* in the periods (from July 2014 to December 2015), from the jugular vein using a sterile collection system (Vacutainer^®^, Becton-Dickinson, USA) without anticoagulant. The blood was then transported under refrigeration to the laboratory, where they were centrifuged at 2500 g for 10 min to obtain serum, which was transferred to clean microtubes. The sera sampled were stored at −20°C until serological analysis.

### Animals and risk factors

After acceptation of the horse’s owners, they received a questionnaire asking for: Region/Governorate, habit, breed, gender, age, horse feed (forage, pasture, and concentrated), water source (tap, wells, and water tank), and coat color. A complete clinical examination was performed on each horse.

The study involved a total of 736 horses from 25 governorates: North region (n=363), highlands (n=315), and region south (n=58). All the horses lived in 167 different farms (n=526), 10 racecourses (n=88), and 18 equestrian centers (n=122), including 421 males, 288 females, and 27 gelding. The included horses belonged to different breeds; Barb (n=231), Arab/Barb (n=223), Arabian (n=117), Thoroughbred (n=69), AQPS (French chaser) (n=27), French Saddlebred (n=22), Pony (n=19), Anglo/barb (n=11), Trotter (n=9), Irish Sport Horse (n=3), Anglo/Arabian (n=2), Koninklijke Vereniging Warmbloed Paardenstamboek Nederland (n=2), and Breton (n=1).

The different ages of animals were pooled into four groups [13], one group with an age between 1 and 5 years old (n=154), the second group with horses between 6 and 10 years old (n=377), the third group with horses between 11 and 15 years old (n=164), and the last group with horses >15 years (41). The horses had different colors: Bay (250), Chestnut (226), Grey (194), Roan (41), Seal brown (11), Chestnut roan (7), Piebald (4), Chestnut dun (2), and Buckskin (1).

Domestic and stray cats are in free circulation in all farms, racecourses, and equestrian center of the present study.

### Serological analysis

#### IFAT

An in house IFAT was used to detect antibodies to *T. gondii* in equine sera, as described previously. The antigens used are the tachyzoïtes of *T. gondii* of the stock RH. Twelve-spot IFAT glass slides (International Medical Products, Brussels, Belgium) were coated, air-dried, and stored at −20°C until use. Sera diluted in phosphate-buffered saline (PBS) supplemented (1 g/l) with bovine serum albumin (PBS-BSA) (Sigma-Aldrich, Bornem, Belgium) were deposited in separated wells, and the slides were incubated at 37°C for 30 min in a moist atmosphere. Then, they were gently rinsed with IFAT buffer (0.9% NaCl) and incubated for a further 30 min. Fluorescein labeled affinity-purified rabbit anti-equine IgG (SIGMA, Saint Louis, MO, USA) diluted 1/64 in PBS-BSA was added for 30 min. After a final rinse, the slides were mounted in IFAT-buffer (50% v/v glycerol and 0.9% NaCl) and examined under epifluorescence using a Leitz Laborlux S epifluorescence microscope (Leitz, Wetzlar, Germany) [14]. Each batch of slides included a known positive and a known negative equine control sera. Sera were considered positive if the entire surface of the tachyzoites was fluorescent with a titer ≥1/50 [15]. On microscopic examination, the signal for each sample was classified by a simple grading system; no signal, doubtful (d), positive (+), strong positive (++), and highly positive (+++).

Both the IFAT and the preparation of slides containing *T. gondii* antigens were performed at the Laboratory of Parasitology and Parasitic Disease, University of Liege (Belgium).

#### ELISA

The sera were analyzed using the multi-species ID Screen Toxoplasmosis Indirect lit (IDVET, Montpellier, France) according to the recommendation of the fabricant.

Positive samples from an antigen-antibody combined horseradish peroxidase complex are revealed by a color change by the revealing solution (TMB). The reaction was stopped with 100 µl per well of 0.5 M sulfuric acid and the optical density (OD) was measured at 450 nm and read in a microplate reader BIO-TEK model EL 800. The mean ODs obtained for the positive and negative sera were 1.026±0.338 (n=317) and 0.228±0.096 (n=419).

#### LAT

Toxo-Latex^®^ (SPINRER EACT, S.A. Ctra. Santa Coloma, Spain) was used to reveal the presence of the anti-*T. gondii* antibodies in a qualitative and semi-quantitative way. The test was performed according to the manufacturer’s instructions. Different cutoff points ranging from 1/16 to 1/1024 were used with a titer equal to or higher than 1/16 is regarded as positive. In the present study, the smallest tested titer was 1/16, according to the LAT kit instructions.

### Statistical analysis

Statistical differences in proportions were compared using the Chi-square test (Yates corrected) or Fisher’s exact test. The strength of the association between serological status and the epidemiological factors assessed here was estimated by computing the odds ratios (OR).

Furthermore, the agreement between ELISA, IFAT, and LAT was assessed by calculation of kappa statistic value. Kappa value was interpreted as Petrie and Watson (1999): k ≤0.20 without consistency, 0.21 ≤ k ≤0.40 poor agreement, 0.41≤ k ≤0.60 moderate agreement, 0.61 ≤ k ≤0.80 good agreement, and k>0.80 very good agreement.

Win episcope 2.0, MedCalc, and XLstat softwares were used. The level of statistical significance used was 5%. If the p-values were lower, it was indicated in the text.

## Results

### Seroprevalence per methods

Antibodies to *T*. *gondii* were detected in 178 of 736 horses with IFAT (24.18%), 317 with ELISA (43.07%), and 133 with LAT (18.07%). The prevalence per method was significantly different (p<0.05). The seroprevalence per region/per governorate with the three methods is presented in Table-1.

**Table-1:** Seroprevalence of *Toxoplasma gondii* in different Algerian governorates by IFAT, ELISA, and LAT.

ID-Governorate/region	n	IFAT		ELISA		LAT		p-value
		
		Positive	Seroprevalence % (95% CI)	Positive	Seroprevalence % (95% CI)	Positive	Seroprevalence % (95% CI)	
Total	736	178	24.18 (21.02-27.34)	317	43.07 (39.42-46.72)	133	18.07 (15.23-20.91)	<0.01
Region North								
16-Alger	69	11	15.94 (7.13-24.75)	17	24.64 (14.26-35.02)	9	13.04 (4.93-21.15)	NS
09-Blida	12	7	58.33 (29.87-86.79)	9	75.00 (50-100)	7	58.33 (29.87-86.79)	NS
44-Ain Defla	14	4	28.57 (4.42-52.72)	6	42.86 (16.41-69.31)	1	7.14 (0-20.90)	NS
41-Souk Ahras	10	2	20.00 (0-45.30)	3	30.00 (1.02-58.98)	2	20.00 (0-45.30)	NS
27-Mosta	45	0	0.00 (0-0)	3	6.67 (0-14.11)	0	0.00 (0-0)	NS
13-Tlemncen	45	10	22.22 (9.83-34.61)	19	42.22 (27.49-56.95)	7	15.56 (4.75-26.37)	0.01
31-Oran	23	6	26.09 (7.78-44.40)	17	73.91 (55.60-92.22)	2	8.70 (0-20.45)	<0.01
48-Relizane	63	11	17.46 (7.89-27.03)	26	41.27 (28.86-53.68)	7	11.11 (3.19-19.03)	<0.01
22-Sba	32	4	12.50 (0.81-24.19)	5	15.63 (2.79-28.47)	2	6.25 (0-14.81)	NS
29-Mascara	50	27	54.00 (39.90-68.10)	39	78.00 (66.28-89.72)	23	46.00 (31.90-60.10)	<0.01
Total North	363	82	22.59 (18.20-26.98)	144	39.67 (34.53-44.81)	60	16.53 (12.63-20.43)	<0.01
Highlands								
17-Djelfa	19	9	47.37 (24.46-70.28)	10	52.63 (29.72-75.54)	7	36.84 (14.71-58.97)	NS
03-Laghouat	51	11	21.57 (10.05-33.09)	28	54.90 (40.96-68.84)	9	17.65 (6.97-28.33)	<0.01
28-Msila	15	4	26.67 (3.83-49.51)	5	33.33 (8.99-57.67)	0	0.00 (0-0)	<0.01
19-Setif	33	10	30.30 (14.30-46.30)	17	51.52 (34.12-68.92)	8	24.24 (9.32-39.16)	NS
05-Batna	11	5	45.45 (15.42-75.48)	8	72.73 (45.87-99.59)	5	45.45 (15.42-75.48)	NS
40-Khenchla	10	2	20.00 (0-45.30)	5	50.00 (18.38-81.62)	1	10.00 (0-28.97)	NS
34-Bbouriridj	15	7	46.67 (20.91-72.43)	10	66.67 (42.33-91.01)	5	33.33 (8.99-57.67)	NS
20-Saida	26	6	23.08 (6.55-39.61)	11	42.31 (22.93-61.69)	6	23.08 (6.55-39.61)	NS
14-Tiaret	68	6	8.82 (1.94-15.70)	17	25.00 (14.50-35.50)	6	8.82 (1.94-15.70)	<0.01
32-El-Bayadh	30	7	23.33 (7.89-38.77)	19	63.33 (45.73-80.93)	6	20.00 (5.39-34.61)	<0.01
38-Tissemsilt	20	10	50.00 (27.64-72.36)	12	60.00 (38.09-81.91)	9	45.00 (22.75-67.25)	NS
45-Naama	17	6	35.29 (12.11-58.47)	10	58.82 (34.95-82.69)	5	29.41 (7.31-51.51)	NS
Total Highlands	315	83	26.35 (21.39-31.31)	152	48.25 (42.62-53.88)	67	21.27 (16.66-25.88)	<0.01
Region South								
07-Biskra	13	1	7.69 (0-22.47)	1	7.69 (0-22.47)	0	0.00 (0-0)	NS
30-Ouargala	29	7	24.14 (8.25-40.03)	12	41.38 (23.09-59.67)	4	13.79 (0.98-26.60)	NS
47-Ghardaia	16	5	31.25 (8.07-54.43)	8	50.00 (25-75)	2	12.50 (0-29.04)	NS
Total South	58	13	22.41 (11.46-33.36)	21	36.21 (23.59-48.83)	6	10.34 (2.34-18.34)	<0.01

CI=Confidence Interval of 95%. NS=Not statistically significant difference (p>0.05). IFAT=Indirect fluorescent antibody test, LAT=Latex agglutination test, ELISA=Enzyme-linked immunosorbent assay

The agreement between ELISA and IFAT was considerate as moderate (Kappa=0.59) with a percentage of agreement of 81% (597/636). The agreement between ELISA and LAT was considerate as moderate (Kappa=0.49) with a percentage of agreement of 75% (552/736). The agreement between LAT and IFAT was good (kappa=0.79), with a percentage of agreement of 93% (685/736). This result indicated that LAT and IFAT had similar capabilities in the detection of anti-*T. gondii* antibodies from horse sera (Table-2).

**Table-2:** Agreement Analysis between IFAT, ELISA, and LAT tests.

	IFAT				Statistics
ELISA	Sera	Positive	Negative	Total	Kappa=0.59
	Positive	178	139	317	% agreement=81
	Negative	0	419	419	Relative sensitivity=100%
	Total	178	558	736	Relative sensitivity=75.1%
ELISA	LAT				
	Sera	Positive	Negative	Total	Kappa=0.45
	Positive	133	184	317	% agreement=75
	Negative	0	419	419	Relative sensitivity=ND
	Total	133	303	736	Relative sensitivity=ND
LAT	IFAT				
	Sera	Positive	Negative	Total	Kappa=0.79
	Positive	130	3	133	% agreement=93
	Negative	48	555	419	Relative sensitivity=73%
	Total	178	558	736	Relative sensitivity=99.5%

% of agreement=(Number of positive for both methods+number of negative for both method)/736)*100, Relative sensitivity=(Number of positive for both methods/[number of positive for the reference method+number of negative for the other method])*100=(true positive/[true positive+false negative])*100, Relative specificity=(Number of negative for both methods/[number of negative for the reference method+number of positive for the other method])*100=(true negative/[true negative+false positive])*100. IFAT=Indirect fluorescent antibody test, LAT=Latex agglutination test, ELISA=Enzyme-linked immunosorbent assay

### Risk factors

The association between the seroprevalence and several epidemiological factors was investigated using the Chi-square test (Yates corrected) or Fisher’s exact test (Table-3). For the OR calculations, the significant factors were taken into account.

**Table-3:** General characteristics of the 736 horses studied and seroprevalence of *Toxoplasma gondii* infection.

Category	Examined	Positive	Prevalence %	95%CI	p-value	OR (95%CI)
Total	736	178	24.18	(21.03-27.34)		
Habit						
Equestrian center	122	8	6.56	(2.08-11.04)	0,02	0.21 (0.1-0.44)
Racing course	88	20	22.73	(13.79-31.66)		
Farm	526	150	28.52	(24.58-32.45)		
Age						
1-5 years	154	17	11.04	(5.99-16.09)	<0.001	3.72 (2.64-5.24)
6-10 years	377	98	25.99	(21.09-29.64)		
11-15 years	164	79	48.17	(40.37-55.97)		
years >15	41	25	60.98	(45.74-76.22)		
Sex						
Male	421	102	24.23	(20.05-28.40)	<0.05	0.12 (0.02-0.89)
Female	288	75	26.04	(20.87-31.21)		
Gelding	27	1	3.70	(0-10.97)		
Breed						
Arab	117	33	28.21	(19.88-36.53)	<0.01	1.77 (1.25-2.51)
Barb	231	49	21.21	(15.83-26.59)		
Arab/barb	223	70	31.39	(25.17-37.61)		
Anglo/Arab	2	0	0.00	0		
Anglo/barb	11	4	36.36	(7.36-65.37)		
Thoroughbred	69	10	14.49	(6.02-22.97)		
AQPS	27	8	29.63	(12.05-47.21)		
KWPN	2	0	0.00	0		
Irish	3	0	0.00	0		
Breton	1	0	0.00	0		
French Saddlebred	22	0	0.00	0		
Pony	19	3	15.79	(0-32.52)		
Trotter	9	1	11.11	(0-32.06)		
Colors						
Chestnut	226	55	24.34	(18.63-30.05)	<0.01	NT
Bay	250	64	25.60	(20.08-31.12)		
Grey	194	46	23.71	(17.60-29.82)		
Seal brown	11	4	36.36	(7.36-65.37)		
Chestnut roan	7	1	14.29	(0-40.74)		
Chestnut dun	2	0	0.00	0		
Buckskin	1	0	0.00	0		
Piebald	4	0	0.00	0		
Roan	41	8	19.51	(7.13-31.89)		
Food						
Forage	113	25	22.12	(14.31-29.93)	NS	NT
Pasture	4	1	25.00	(0-68.30)		
For/past	8	3	37.50	(3.27-71.73)		
Concentrated/for	542	132	24.35	(20.67-28.04)		
Conc/past	3	1	33.33	(0-87.77)		
Conc/past/for	59	16	27.12	(15.54-38.69)		
Other/forage	7	0	0.00	0		
Water						
Taps	238	20	8.40	(4.81-12.)	<0.01	
Water tanks	268	81	30.22	(24.61-35.83)		0.2 (0.12-0,33)
Wells	211	73	34.60	(28.05-41.15)		
Water tanks/wells	19	4	21.05	(2.35-39.76)		
Governorate						
Northern Algeria	363	82	22.59	(18.20-26.98)		
Highlands	315	83	26.35	(21.39-31.31)	NS	NT
South	58	13	22.41	(11.46-33.36)		

NS=Not significant (p≥0.05) NT=Not tested, OR=Odds ratio, CI=Confidence interval of 95%. KWPN=Koninklijke Vereniging Warmbloed Paardenstamboek Nederland

This risk factor analysis led to some conclusions. The horses from equestrian centers were significantly less positive (6.56%, 95 CI: 2.08-11.04, p<0.05). The facts to belong to an equestrian center are a protective factor against *T*. *gondii* infection (OR significantly lower than 1).

There was an increase in seroprevalence with the age of the horses. The horses older and younger than 10 years were compared, and the OR indicated that the belonging to older group is a risk factor to be positive in IFAT (OR significantly upper than 1).

The geldings were less contaminated than the males or the females (3.70%, 95% CI: 0-10.97, p<0.05). The gelding appears to be protected from *T. gondii* infection (OR significantly less than 1).

There was also an association between the genetic of the horse (Breed, and Coat color) and the seroprevalence with several breeds without seropositive samples and some with higher seroprevalence.

Arab/barb and Anglo/barb horses were significantly more susceptible to *T. gondii* infection (OR significantly higher than 1) in comparison to other breeds.

The type of food is not relevant, but the source of water is important since tap water is clearly associated with lower prevalence (8.40%, 95 CI: 4.81-12, p<0.01). The consumption of tap water is a protective factor for *T. gondii* infection (OR significantly lower than 1).

There is no evident relationship between the region of the horse and the seroprevalence. To investigate more deeply this point, an analysis per governorate was performed (Table-4). The results indicated that the variation of seroprevalence is higher within the regions (it means between the governorates of the same region) than between the regions.

**Table-4:** Seroprevalence of *Toxoplasma gondii* in different Governorate of Algeria by IFAT.

Region ID/governorate	Analyzed	Positive	Seroprevalence % (95% CI)	p-value
Total	736	178	24.18 (21.02-27.34)	
North				
16-Alger	69	11	15.94 (7.13-24.75)	<0.01
09-Blida	12	7	58.33 (29.87-86.79)	
44-Ain Defla	14	4	28.57 (4.42-52.72)	
41-Souk Ahras	10	2	20.00 (0-45.30)	
27-Mosta	45	0	0.00 (0-0)	
13-Tlemncen	45	10	22.22 (9.83-34.61)	
31-Oran	23	6	26.09 (7.78-44.40)	
48-Relizane	63	11	17.46 (7.89-27.03)	
22-Sba	32	4	12.50 (0.81-24.19)	
29-Mascara	50	27	54.00 (39.90-68.10)	
Total	363	82	22.59 (18.20-26.98)	
Highlands				
17-Djelfa	19	9	47.37 (24.46-70.28)	<0.01
03-Laghouat	51	11	21.57 (10.05-33.09)	
28-Msila	15	4	26.67 (3.83-49.51)	
19-Setif	33	10	30.30 (14.30-46.30)	
05-Batna	11	5	45.45 (15.42-75.48)	
40-Khenchla	10	2	20.00 (/-45.30)	
34-Bbouriridj	15	7	46.67 (20.91-72.43)	
20-Saida	26	6	23.08 (6.55-39.61)	
14-Tiaret	68	6	8.82 (1.94-15.70)	
32-El-Bayadh	30	7	23.33 (7.89-38.77)	
38-Tissemsilt	20	10	50.00 (27.64-72.36)	
45-Naama	17	6	35.29 (12.11-58.47)	
Total	315	83	26.35 (21.39-31.31)	
South				
07-Biskra	13	1	7.69 (0-22.47)	NS
30-Ouargala	29	7	24.14 (8.25-40.03)	
47-Ghardaia	16	5	31.25 (8.07-54.43)	
Total	58	13	22.41 (11.46-33.36)	

NS=Not significant (p≥0.05), CI=Confidence interval, IFAT=Indirect fluorescent antibody test

## Discussion

*T. gondii* is a zoonotic parasite responsible for diseases in animals and humans worldwide. The surveillance of such a pathogen is important for epidemiological purposes [16].

The estimated seroprevalence of *T. gondii* in horses can vary with the serological test used. In the present survey, the serum samples were tested by three different methods. The prevalence recorded is 24.18% by IFAT, 18.07% by LAT, and 43.07% by ELISA. A relatively large number of studies report on the seroprevalence of antibodies against *T. gondii* in horses [5]. The seropositivity in Italy was 3% by IFAT [17]; in Hakkari, Eastern region of Turkey, the seropositivity was 13.5% and 28.3% with indirect hemagglutination and Sabin–Feldman dye test, respectively [18]. The Czech Republic was with 24% by LAT [19] and Tunisia was with 17.7% by MAT [20]. In Algeria, 26% by MAT [21], these are the only results that we have for Algeria and join the results of our study carried out on the national territory. It is difficult to compare the seroprevalence since the samples are different and the used methods also. Nevertheless, it is clear that the Algerian horses were frequently in contact with the parasite.

In this study, we compared the sensitivities of three methods for the detection of antibodies against *T. gondii* in naturally infected horses using commercial kits ELISA and LAT, in comparison to in-house serological reference test (IFAT) [22]. Indeed, IFAT is considered as a gold standard in serodiagnostic of *Toxoplasma* infection in several animal species [14]. This test detects antibodies directed to antigens present on the cell surface of the tachyzoites; such antigens, in the *Apicomplexa*, are more specific than intracellular ones [23,24]. This feature combined with very little cross-reactivity with related protozoa, leads IFAT to be considered almost perfectly specific. On the other hand, IFAT sensitivity was already assessed [25]. ELISA used is the multi-species ID Screen^®^ Toxoplasmosis Indirect kit (IDVET, Montpellier) for the detection of antibodies against the *Toxoplasma* P30 protein. This kit was used in horses in a single study in New Caledonia [26]. The sera were also tested with LAT using Toxo-Latex^®^. Because ELISA and IFAT enable the detection of the *T. gondii* IgG class antibodies only, whereas LAT enables detecting antibodies of both classes – IgG and IgM, then hypothetically LAT should be an optimal test to screening study [23]. An agreement analysis between three methods showed moderate agreement (Kappa 0.59) between ELISA and IFAT. An attempt to use recombinant P30 antigen was effective in detecting antibodies to *T. gondii* in experimentally infected pigs [27]. However, sera from naturally infected pigs tested with P30 antigen indicated a sensitivity of the test reduced to an unacceptable level [28].

A substantial agreement (Kappa 0.79) is obtained between LAT and IFAT. This result indicated that LAT and IFAT had similar capabilities in the detection of anti-*T. gondii* antibodies from horse sera. A preliminary study has shown that LAT used to detect *Toxoplasma* antibodies in sera of cats revealed better sensitivity and specificity [23]. This indicates that commercial test LAT could be used for the examination of other animal species.

Based on the results of this study, the LAT demonstrated similar, good correlation in the detection of serum antibodies to *Toxoplasma* in horses (Cohen’s Kappa statistic K around 0.8) in comparison to reference IFAT. The test ELISA had poor specificity when compared with the other tests. However, some authors considered the insensitivity of LAT in the detection of *Toxoplasma* antibodies in pigs [29]. Indeed, the LAT showed a relative sensitivity of 73% in our study but the relative sensitivity was 99.5%. Therefore, the LAT has a high capability to identify a negative serum but a lower capacity to detect a positive serum. The ELISA showed exactly opposite results with high sensitivity (100%), meaning that all the sera positive in IFAT were also positive in ELISA, but low specificity (75%) indicated that the ELISA detected false positive sera if the IFAT is taken as the reference method.

Therefore, since the IFAT is more fastidious than the commercial methods, as two-step strategies can be proposed. The first screening by ELISA was done then by the LAT test for the detection of true positives and false positives. In this way, the capability to detect a positive sample of the ELISA and the capability of the LAT to detect negative samples were used.

When evaluating the different risk factors for a significant relationship with the presence of anti-Toxoplasma antibodies in horse sing logistical regression analysis, we revealed that sex, age, breed, colors, activity, and water, but not governorate and food, are a significant risk factor for *T. gondii* infection in horses across Algeria.

In the case of age, the result was reinforced by the fact that seroprevalence of *T. gondii* in older horses (year >10) was statistically higher than that in younger horses (1≤ year ≤5). Effectively the result shows a prevalence of 60.98% in older horses versus 11.04% for horses between 1 and 5 years, 25.99% for horses between 6 and 10 years, and 48.17% for horses between 11 and 15 years. These findings suggest that horses can be horizontally infected with *T. gondii* by ingestion of food or water contaminated with oocysts rather than by transplacental infection. The risk of human infection should not be dismissed mainly because a higher seroprevalence was found in older animals that are mostly used for human consumption after retirement. These results are in accordance with the result in Northwestern China [15], Tunisia [20], and Soudan [30]. All found a seroprevalence in the older horses, significantly higher than those of young horses. In contrary, recent studies in other countries showed that there is no significant difference in the seroprevalence of *T. gondii* between age classes in horses; Turkey [18], Mexico [10], Southwestern of China [31], Southern Spain [32], Portugal [33], Korea [34], and Greece [35].

In addition to this difference between the age groups, a difference in the subgroups of females, males, and gelding was also significant. Indeed, the female horses had a significantly higher seroprevalence (26.04%), than the male (24.23%), and gelding (3.70%). It also suggests that female horses are more sensitive to the infection by the parasite than male and gelding, which is the same result that reported in a previous study in Egypt [36]. Similar results were observed in Spain [32], North West Algeria [21], and Xinjiang, Northwestern China [37], but in contrast to our work, their difference was not shown to be statistically significant.

For results on the animal breed, we found a correlation between toxoplasma infection and different horse breeds. Anglo-barb and Arab-barb horses (Arab crossing with barb) seem to be the most sensitive breed for the acquisition of the infection. Work in Tunisia [20] had found a significant difference between seropositivity and breed but citing Arab as the most sensitive. On the other hand, work in a region in Northwestern Algeria [21] showed no significant difference between seropositivity and age, gender, and breed of horses.

For results on the horse’s colors, it is the 1^st^ time that a correlation has been reported between toxoplasmosis infection and different horse colors. There is a significant difference between seropositivity and horse color. Indeed it seems that seal brown horses are the most sensitive for the acquisition of the infection. In addition, the type of activity of equids had a significant effect on the presence of Toxoplasma infection. Our study suggests an influence of the genetic constitution of the horses on the susceptibility to *T. gondii* infection.

The seroprevalence of horses living on farms 28.52% (150/526) is much higher than that of horses living in equestrian centers 6.56% (8/122). This can be explained by the poor hygiene quality of the farms compared with those at the equestrian centers. This also means that athletic horses have lower seropositivity than those who do not train often these results are consistent with those of Greece [35] where higher seroprevalence was confirmed in horses living on farms. This also points to the higher infection risk to humans as they consume horse farm meat.

In our study, cats were in free circulation on farms, equestrian centers, and racecourses. This almost permanent presence of cats represents a risk factor as was described in 2016 in Brazil by reporting that as evidence, this permanent presence of the cats can be at the origin of seroprevalence in horses, and attests that cats are considered risk factors for toxoplasmosis in many species, and noted that the infection was due to the climate, type of farming (extensive, semi-intensive, or intensive), feeding, water supply and especially, to the presence of cats in the properties [38].

This is the first study that takes into consideration the type of watering of horses. The different watering facilities showed that there was a significant difference between *T. gondii* seropositivity and drinking water. Drinking from wells and water tanks lead to higher seroprevalence than tap water. The water source seems to affect the horse habitat risk factor as water in the equine centers comes from the tap while that of the farms comes directly from wells and/or water tanks. Food did not significantly affect the seroprevalence of *T. gondii*.

## Conclusion

This is the first epidemiological survey carried out on more than 20 Algerian governorates to examine the seroprevalence of *T. gondii* in horses. The use of three serological methods (IFAT, LAT, and ELISA) for the detection of *T. gondii* allowed us not only to bring some elements of comparisons but also to have a seroprevalence to *T. gondii* in the horse in Algeria. Although the seroprevalence is lower than in other countries such as in Riyadh Province, Saudi Arabia [9], or in Hakkari, Eastern region of Turkey [18], we have found that *T. gondii* was present in horses in almost all regions. Since horse meat is mainly consumed by people in convalescence, epidemiological investigations are needed to control human infection. Horses are probably infected horizontally by *T. gondii* by ingesting food or water contaminated with oocysts. Prevention of acquired infection of these environmental factors is considered essential for the eradication or control of *T. gondii*, and as well as the development of a reliable and specific method for the detection of *T. gondii* in horses would be interesting to control this zoonosis.

## Authors’ Contributions

SFO, BC, and FG designed the study. SFO, FG, ST, and SYD wrote the manuscript and collected data. SFO collected the samples. SFO, NA, BC, and FG carried out the laboratory work. FG and ST performed statistical analysis. SFO, FG, ST, and BC analyzed the data. SFO and AL reviewed the manuscript. All authors read and approved the final manuscript.

## References

[1] Hanafiah M, Nurcahyo R.W, Yuniar R.S, Prastowo J, Hartati S, Sutrisno B, Aliza D (2017). Detection of *Toxoplasma gondii* in cat's internal organs by immunohistochemistry methods labeled with-[strept] avidin-biotin. Vet. World.

[2] Motarjemi Y, Moy G, Todd E (2014). Encyclopedia of Food Safety 2^nd^ed.

[3] Schlüter D, Däubener W, Schares G, Gross U, Pleyer U, Lüder C (2014). Animals are key to human toxoplasmosis. Int. J. Med. Microbiol.

[4] Masatani T, Takashima Y, Takasu M, Matsuu A, Amaya T (2016). Prevalence of anti *Toxoplasma gondii* antibody in domestic horses in Japan. Parasitol. Int.

[5] Stelzer S, Basso W, Silván J.B, Ortega-Mora L.M, Maksimov P, Gethmann J, Conraths F.J, Schares G (2019). *Toxoplasma gondii* infection and toxoplasmosis in farm animals:Risk factors and economic impact. Food Waterborne Parasitol.

[6] Tonouhewa A.B.N, Akpo A, Adoligbe C, Hounmanou Y.G, Assogba M.N, Youssao I, Yessinou E, Farougou S (2017). *Toxoplasma gondii* infection in meat animals from Africa:Systematic review and meta-analysis of sera-epidemiological studies. Vet. World.

[7] Armand B, Solhjoo K, Kordsooli M.S, Davami M.H (2016). Toxoplasma infection in sheep from south of Iran monitored by serological and molecular methods;risk assessment to meat consumer. Vet. World.

[8] Alvarado-Esquivel C, Rodríguez-Peña S, Villena I, Dubey J.P (2012). Seroprevalence of *Toxoplasma gondii* infection in domestic horses in Durango state, Mexico. J. Parasitol.

[9] Alanazi A.D, Alyousif M.S (2011). Prevalence of antibodies to *Toxoplasma gondii* in horses in Riyadh Province, Saudi Arabia. J. Parasitol.

[10] Karatepe B, Babür C, Karatepe M, Kılıç S (2010). Seroprevalence of toxoplasmosis in horses in Niğde Province of Turkey. Trop. Anim. Health.

[11] Official Journal of the People's Democratic Republic of Algeria (2010). Law No 10-02 of 16 Rajab 1431 Corresponding to June 29 2010, Approving the National Plan of Spatial Planning No. 61.

[12] Thrusfield M (2007). Veterinary Epidemiology 3^rd^ed.

[13] Xing H, Xu L, Song X, Li X, Yan R (2018). Seroprevalence of *Toxoplasma gondii* and *Trichinella spiralis* in horses in Xinjiang, Northwestern China. J. Equine Vet. Sci.

[14] Langoni H, Da Silva A.V, Pezerico S.B, De Lima V.Y (2007). Utilization of modified agglutination test and indirect immunofluorescent antibody test for the detection of *Toxoplasma gondii* antibodies in naturally exposed horses. Braz. J. Vet. Res. Anim. Sci.

[15] Machacova T, Bartova E, Di Loria A, Sedlak K, Mariani U, Fusco G, Fulgione D, Veneziano V, Dubey J.P (2014). Seroprevalence of *Toxoplasma gondii* in donkeys (*Equus asinus*) in Italy. J. Vet. Med. Sci.

[16] Dubey J.P, Porterfield M.L (1986). Toxoplasma-like sporozoa in an aborted equine fetus. J. Am. Vet. Med. Assoc.

[17] Bártová E, Machačová T, Sedlák K, Budíková M, Mariani U, Veneziano V (2015). Seroprevalence of antibodies of *Neospora* spp *Toxoplasma gondii* in horses from Southern Italy. Folia Parasitol.

[18] Göz Y, Babür C, Aydin A, Kiliç S (2007). Seroprevalence of toxoplasmosis, brucellosis and listeriosis in horses in Hakkari, eastern region of Turkey. Rev. Méd. Vét.

[19] Bártová E, Sedlák K, Syrová M, Literák I (2010). *Neospora* spp and *Toxoplasma gondii* antibodies in horses in the Czech Republic. Parasitol. Res.

[20] Boughattas S, Bergaoui R, Essid R, Aoun K, Bouratbine A (2011). Seroprevalence of *Toxoplasma gondii* infection among horses in Tunisia. Parasit. Vectors.

[21] Mohamed-Cherif A, Ait-Oudhia K, Khelef D (2015). Detection of anti-*Toxoplasma gondii* antibodies among horses (*Equus caballus*) and donkeys (*Equus asinus*) in Tiaret province, Northwestern Algeria. Rev. Méd. Vét.

[22] Garcia J.L, Navarro I.T, Vidotto O, Gennari S.M, Machado R.Z, Da Luz Pereira A.B, Sinhorini I.L (2006). *Toxoplasma gondii*:Comparison of a rhoptry-ELISA with IFAT and MAT for antibody detection in sera of experimentally infected pigs. Exp. Parasitol.

[23] Sroka J, Cencek T, Ziomko I, Karamon J, Zwoliński J (2008). Preliminary assessment of Elisa, Mat, and Lat for detecting *Toxoplasma gondii* antibodies in pigs. Bull. Vet. Inst. Pulawy.

[24] Björkman C, Uggla A (1999). Serological diagnosis of *Neospora caninum* infection. Int. J. Parasitol.

[25] Dubey J.P, Hattel A.L, Lindsay D.S, Topper M.J (1988). Neonatal *Neospora caninum* infection in dogs:Isolation of the causative agent and experimental transmission. J. Am. Vet. Med. Assoc.

[26] Roqueplo C, Halos L, Cabre O, Davoust B (2011). *Toxoplasma gondii* in wild and domestic animals from New Caledonia *Parasite*.

[27] Gamble H.R, Andrews C.D, Dubey J.P, Webert D.W, Parmley S.F (2000). Use of recombinant antigens for detection of *Toxoplasma gondii* infection in swine. J. Parasitol.

[28] Gamble H.R, Dubey J.P, Lambillotte D.N (2005). Comparison of a commercial ELISA with the modified agglutination test for detection of *Toxoplasma* infection in the domestic pig. Vet. Parasitol.

[29] Dubey J.P, Thulliez P, Powell E.C (1995). *Toxoplasma gondii* in Iowa sows:Comparison of antibody titers to isolation of *T*.*gondii* by bioassays in mice and cats. J. Parasitol.

[30] Ishag M.Y, Majid A.M, Abuobeida M.M (2014). Studies on toxoplasmosis in horses in Khartoum state-Sudan. J. Vet. Med. Anim. Prod.

[31] Miao Q, Wang X, She L.N, Fan Y.T, Yuan F.Z, Yang J.F, Zhu X.Q, Zou F.C (2013). Seroprevalence of *Toxoplasma gondii* in horses and donkeys in Yunnan Province, Southwestern China. Parasit. Vectors.

[32] García-Bocanegra I, Cabezón O, Arenas-Montes A, Carbonero A, Dubey J.P, Perea A, Almería S (2012). Seroprevalence of *Toxoplasma gondii* in equids in Southern Spain. Parasitol. Int.

[33] Lopes A.P, Sousa S, Dubey J.P, Ribeiro A.J, Silvestre R, Cotovio M, Schallig H.D.F, Cardoso L, Cordeiro-da-Silva A (2013). Prevalence of antibodies to *Leishmania infantum* and *Toxoplasma gondii* in horses from the north of Portugal. Parasit. Vectors.

[34] Lee S.H, Lee S.E, Seo M.G, Goo Y.K, Cho K.H, Cho G.J, Kwon O.D, Kwak D, Lee W.J (2014). Evidence of *Toxoplasma gondii* exposure among horses in Korea. J. Vet. Med. Sci.

[35] Kouam M.K, Diakou A, Kanzoura V, Papadopoulos E, Gajadhar A.A, Theodoropoulos G.A (2010). Seroepidemiological study of exposure to *Toxoplasma, Leishmania, Echinococcus* and *Trichinella* in equids in Greece and analysis of risk factors. Vet. Parasitol.

[36] Haridy F.M, Shoukry N.M, Hassan A.A, Morsy T.A (2009). ELISA-seroprevalence of *Toxoplasma gondii* in draught horses in Greater Cairo, Egypt. J. Egypt. Soc. Parasitol.

[37] Wang J.L, Zhou D.H, Chen J, Liu G.X, Pu W.B, Liu T.Y, Qin S.Y, Yin M.Y, Zhu X.Q (2015). The prevalence of antibodies to *Toxoplasma gondii* in horses in Changji Hui autonomous prefecture, Xinjiang, Northwestern China. Braz. J. Vet. Parasitol.

[38] Cazarotto C.J, Balzan A, Grosskopf R.K, Boito J.P, Portella L.P, Vogel F.F, Fávero J.F, Cucco D.C, Biazus A.H, Machado G, Da Silva A.S (2016). Horses seropositive for *Toxoplasma gondii Sarcocystis* spp and *Neospora* spp.:Possible risk factors for infection in Brazil. Microb. Pathog.

